# Persistent Hiccups as an Atypical Presentation of SARS-CoV-2 Infection: A Systematic Review of Case Reports

**DOI:** 10.3389/fneur.2022.819624

**Published:** 2022-04-04

**Authors:** Panagiotis Giannos, Konstantinos Katsikas Triantafyllidis, Georgios Geropoulos, Konstantinos S. Kechagias

**Affiliations:** ^1^Department of Life Sciences, Faculty of Natural Sciences, Imperial College London, London, United Kingdom; ^2^Society of Meta-Research and Biomedical Innovation, London, United Kingdom; ^3^Department of Nutrition and Dietetics, Musgrove Park Hospital, Taunton & Somerset NHS Foundation Trust, Taunton, United Kingdom; ^4^Department of General Surgery, University College London Hospitals, NHS Foundation Trust, London, United Kingdom; ^5^Department of Metabolism, Digestion and Reproduction, Faculty of Medicine, Imperial College London, London, United Kingdom; ^6^Department of Obstetrics and Gynaecology, Chelsea and Westminster Hospital NHS Foundation Trust, London, United Kingdom

**Keywords:** coronavirus, COVID-19, hiccups, singultus, hiccoughs, systematic review, SARS-CoV-2

## Abstract

Symptoms, such as fever, dry cough, dyspnoea, and respiratory distress, are commonly described in patients infected with Severe Acute Respiratory Syndrome Coronavirus 2 (SARS-CoV-2). Recently, a growing number of cases pertained to persistent hiccups have been reported by SARS-CoV-2 infected patients. The aim of this systematic review was to screen the current literature and provide a summary of the reported cases of SARS-CoV-2 infected patients presenting with persistent hiccups. According to Preferred Reporting Items for Systematic Reviews and Meta-Analyses (PRISMA) guidelines, PubMed, Scopus, and Web of Science databases were searched from inception until October 2021. Case reports or case series that provided a separate clinical description for patients with presenting complaints of persistent hiccups before or after COVID-19 diagnosis were retrieved. The critical appraisal checklist for case reports provided by the Joanna Briggs Institute (JBI) was employed to evaluate the overall quality of the eligible studies. We identified 13 eligible studies that included 16 hospitalized COVID-19 patients who complained of persistent hiccups. The mean duration of hiccups was 4.6 days reported in 88% (14/16) patients. Hypertension was the most common comorbidity present in 50% (8/16) of patients followed by diabetes mellitus (4/16). Moreover, 44% (7/16) of patients received only one medication for managing the hiccups with metoclopramide (5/16) followed by chlorpromazine and baclofen (4/16) used as primary treatment. Equally, 44% of patients (7/16) received dexamethasone followed by azithromycin (5/16), ivermectin (4/16), and ceftriaxone (4/16) for managing the infection from SARS-CoV-2. The majority of patients (14/16) improved after initiation of treatment. Persistent hiccups are possibly a rare symptom that clinicians may expect to encounter in patients infected with SARS-CoV-2. Although there is not ample proof to propose causation, increased awareness about the diversity of presentations of SARS-CoV-2 infection could be crucial in the early recognition of the disease.

## Introduction

In December 2019, an atypical case of viral pneumonia caused by a novel coronavirus called Severe Acute Respiratory Syndrome Coronavirus 2 (SARS-CoV-2) was discovered in Wuhan, Hubei Province of China (1, 2). Since then, the disease (COVID-19) has spread worldwide with currently more than 250,000,000 confirmed cases reported.

Patients infected with SARS-CoV-2 have a characteristic clinical presentation that includes fever, dry cough, progressive dyspnoea, and respiratory distress (3). The disease caused by this virus may also confer non-respiratory manifestations, including gastrointestinal symptoms, such as vomiting, diarrhea, and abdominal pain (4, 5). Beyond the most commonly presenting symptoms, a diverse range of complaints, including myalgia, dizziness, and headache, have been reported by patients (6, 7). More recently, there is a growing number of complaints pertained to persistent hiccups, which have been documented in patients with COVID-19 (8).

Hiccups, also known as singultus or hiccoughs, are involuntary reflex movements of diaphragmatic contractions that lead to the sudden closure of the glottis and the abrupt termination of inspiration (9, 10). Most commonly, hiccups are recognized as acute and are often of short duration, lasting less than 48 h (9–11). However, persistent hiccups or those longer than 48 h are often associated with cardiac or gastrointestinal disorders, which potentially underlie a manifestation of a major pathology (9–11).

Persistent hiccups can be burdensome to patients with a great impact on quality of life and often difficult to manage (12, 13). If left untreated, these can lead to sleep disturbances, physical exhaustion, and even depression (12, 13). To date, persistent hiccups as an atypical symptom of SARS-CoV-2 infection have not been considered. The aim of our systematic review was to comprehensively screen all currently available literature and provide a detailed summary of all the reported cases of SARS-CoV-2 infected patients presenting with persistent hiccups.

## Materials and Methods

This review was composed on the basis of the “Preferred Reporting Items for Systematic Reviews and Meta-Analyses” (PRISMA) guidelines (14).

### Literature Search

Two independent reviewers (PG and KKT) screened the PubMed, Scopus, and Web of Science databases from inception until October 2021, using the following terms: “(COVID 19) OR (SARS-COV2) OR (Coronavirus) OR (2019-nCoV) AND (hiccups) OR (hiccoughs) OR (singultus) OR (synchronous diaphragmatic flutter)”. A manual search of references cited in eligible publications, was also performed for undetected studies. No restrictions pertained to study design and geographic origin were applied to our search and all discrepancies were resolved by a third investigator (GG).

### Eligibility Criteria

We included case reports or case series that provided a separate clinical description for patients with presenting complaints of persistent hiccups before or after the COVID-19 diagnosis. Non-English articles, review articles, abstracts and non-peer-reviewed sources were classified as ineligible for inclusion. Studies with *in vitro* and animal models were also excluded.

### Data Extraction and Handling

We extracted individual patient data from eligible studies and collected the following information: gender, age, reported comorbidities and cardinal COVID-19 symptoms, onset of hiccups before or after COVID-19 diagnosis, duration of hiccups and outcome, use of laboratory tests, and treatment regime. Two authors (PG and KKT) conducted the data extraction independently and any disagreements were discussed and resolved by a third investigator (KSK).

### Quality Assessment

The critical appraisal checklist for case reports provided by the Joanna Briggs Institute (JBI) was employed to evaluate the overall quality of the included studies (15). The assessment was ensued based on the reporting of 8 different elements namely, patient demographics, medical history, health status, physical examination and diagnosis, concomitant therapies, post-intervention health status and drug administration reaction interface. The studies were scored either based on “Yes”, “No”, “Unclear or Not/Applicable” depending on the availability of information for every element.

## Results

### Characteristics of Studies

**Figure 1:**
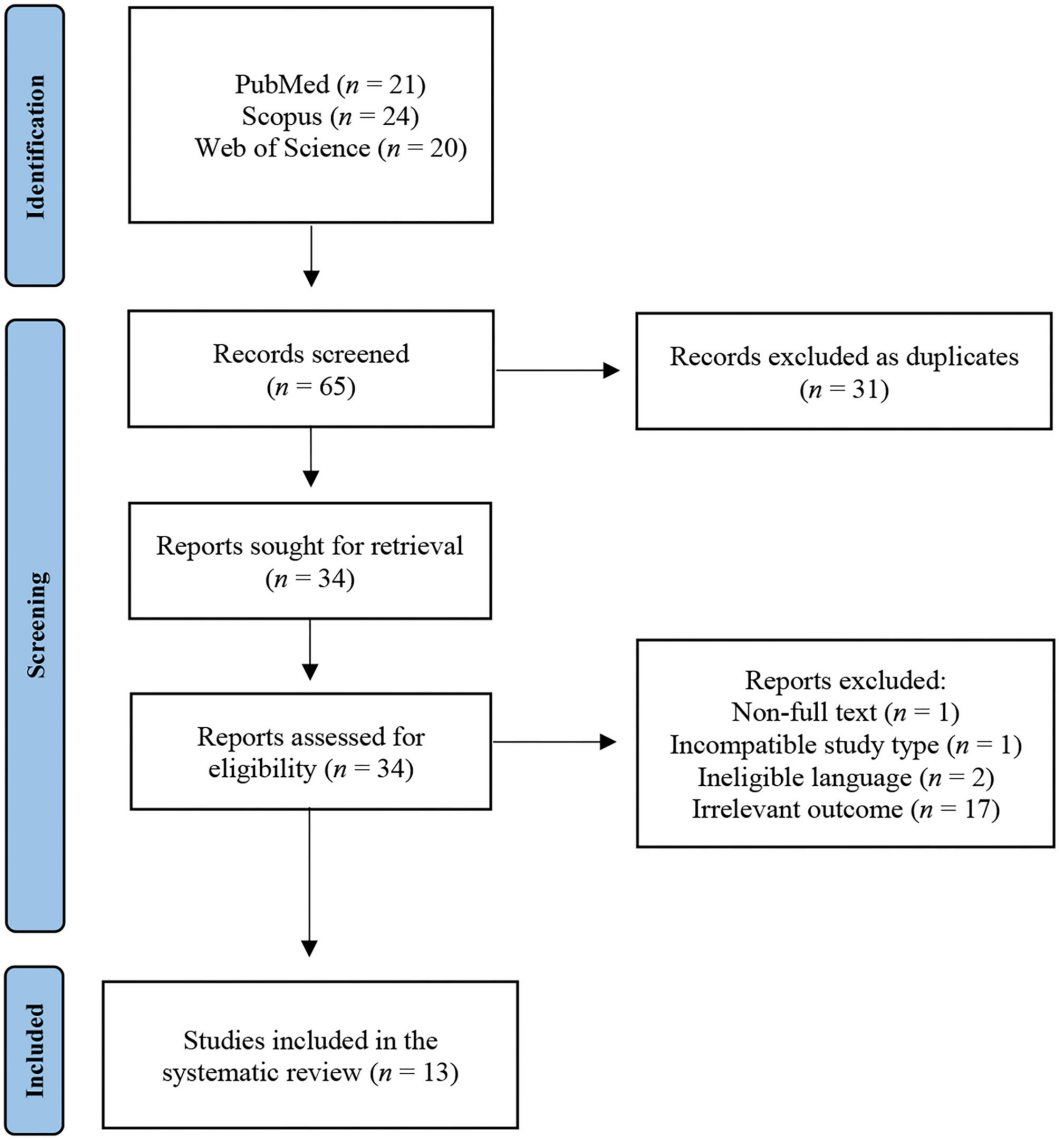
Flowchart of the employed literature search.

Our initial search of the literature resulted in 65 studies. After removal of 31 duplicates, 34 unique studies were retrieved. Upon screening of title and abstract, 17 studies containing irrelevant outcomes, two written in non-English language, one with incompatible study design, and one with no full-text availability, were excluded. Overall, 13 studies were regarded as eligible for inclusion in the systematic review (Figure 1). Eleven of the studies were case reports and two were case series. Six of the studies originated from the Americas, five from Asia, and two from Africa.

The majority of the included patients (14/16) had hiccups prior to COVID-19 diagnosis, with only two developing symptoms afterward.

We retrieved a total of 16 cases of COVID-19 patients with presenting complaints of persistent hiccups. The majority of the included patients (14/16) had hiccups before COVID-19 diagnosis, while in two symptoms developed thereafter. All patients were male apart from a single female, with a mean age of 56 years. Hypertension was the most common comorbidity present in 50% (8/16) of patients followed by diabetes mellitus in 25% (4/16) of patients. Laboratory measurements were available for all patients with the most common characteristic being elevated C-reactive protein, present in 56% (9/16) of patients. A drop in oxygen saturation below 95% was experienced in six patients and four patients were found with attenuated serum sodium levels below 135 mmol/L. Findings of interstitial pneumonia compatible with COVID-19 from chest X-ray or computed tomography scans were present in all the patients. Symptoms of fever along with hiccups were reported in six patients, while 44% (7/16) were free of typical COVID-19 symptoms. The mean duration of hiccups was 4.6 days reported in 88% (14/16) patients. In two studies hiccups lasting more than 2 days were reported, but without any mention of the exact duration before seeking medical advice. For managing the hiccups, 44% of patients (7/16) received metoclopramide (5/16) as medication followed by chlorpromazine and baclofen (4/16), given as primary treatment. Furthermore, 44% of patients (7/16) received dexamethasone followed by azithromycin (5/16), ivermectin (4/16) and ceftriaxone (4/16) for managing the infection from SARS-CoV-2. A larger proportion of patients (14/16) improved after initiation of treatment, while only two showed no signs of improvement (Table 1).

**Table 1:** Clinical characteristics of patients in the included studies.

**Author, year (country)**	**Age (gender)**	**Comorbidities**	**Laboratory findings**	**COVID-19 symptoms**	**Hiccups duration**	**Outcome**	**Therapy**
Ali, 2021 (Kenya) (16)	65 (M)	•Diabetes •Hypertension	•↑ CRP •↑ D-dimer •↓ Lymphocytes •94% SPO2 (hypoxemia)	NA	7 days	Symptoms improved	•Baclofen
Alvarez-Cisneros, 2021 (Mexico) (17)	48 (M)	NA	•↑ BG •↓ Lymphocytes •↓ PLT •↓ WBCs •93% SPO2 (hypoxemia)	NA	4 days	No improvement	•Magaldrate/dimeticone •Metoclopramide •Omeprazole •Ondansetron
Atiyat, 2021 (USA) (18)	61 (M)	•Hypertension	•↑ D-dimer •↑ Ferritin •↑ Lactic acid •↑ LDH •↑ Procalcitonin	•Chest pain •Subjective fever	2 days	Symptoms improved	•Azithromycin •Ceftriaxone •Dexamethasone •Metoclopramide
Bakheet, 2021 (Egypt) (19)	48 (M)	•Hypertension	•↑ CRP •↑ Ferritin •↑ LDH •↑ RR	•Fever •Sore throat	7 days	Symptoms improved	•Antipyretics •Ascorbic acid •Azithromycin •Baclofen •Ceftriaxone •Domperidone •Hydroxychloroquine •Oseltamivir •Prophylactic anticoagulation •Proton pump inhibitors •Zinc
Chiquete, 2021 (Mexico) (20)	62 (M)	•Hypertension •Hypercholesterolemia •Hypertriglyceridemia •Obesity •Impaired fasting glucose •Mild vascular cognitive impairment	•↑ CRP •↑ D-dimer •↑ Ferritin •↑ LDH •92% SPO2 (hypoxemia)	•Cough fever •Mild dyspnea	5 days	Symptoms improved after 3 days	•Azithromycin •Dexamethasone •Enoxaparin •Hydroxychloroquine •Ivermectin •Levomepromazine •Levosulpiride
Dorgalale, 2020 (Iran) (21)	52 (M)	•Diabetes •Congenital factor V deficiency	•↑ ALP •↑ ALT •↑ AST •↑ CRP •↑ FBS •↑ RBCs	NA	> 2 days	Symptoms improved after 5 days	•Chlorpromazine •Metoclopramide •Vitamin B12 and B complex •Vitamin C
Ikitimur, 2021 (Turkey)^*^ (22)	60 (M)	NA	•↑ ALT •↑ CRP •↑ Ferritin •↑ Neutrophils •↑ RR •↑ Urea •↓ Lymphocytes •↓ Troponin	NA	3 days	Symptoms improved after 12 hours	•Azithromycin •Chlorpromazine •Dexamethasone •Favipiravir
	68 (M)	•Hypertension •Childhood poliomyelitis	•↑ CRP •↑ Neutrophils •↑ RR •↑ Urea •↓ Hb •↓ Lymphocytes	NA	4 days	Symptoms improved after 3 days	•Ceftriaxone •Chlorpromazine •Enoxaparin •Favipiravir •Metoclopramide •Plaquenil
Karampoor, 2021 (Iran)^*^ (23)	58 (M)	NA	•↑ Ferritin •↑ LDH •↑ RR •↑ Urea •↓ PLT •90% SPO2 (hypoxemia)	•Dry cough •Fever •Myalgia	6 days	Symptoms improved	•Dexamethasone •Diphenhydramine •Famotidine •Prednisolone •Remdesivir •Vitamin C •Zinc
Prince, 2020 (USA) (8)	68 (M)	•Diabetes •Hypertension •Coronary artery disease	•↓ Cl •↓ Na •↓ PLT •↓ WBCs	•Fever •Tachycardia	4 days	Symptoms improved	•Azithromycin •Ceftriaxone •Hydroxychloroquine
Sangamesh, 2021 (India) (24)	72 (M)	•Hypertension Diabetes	•↑ CRP •↑ Ferritin •↑ LDH •↑ Urea •↓ Na •92% SPO2 (hypoxemia)	•NA	5 days	Symptoms improved after 2 days	•Acebrophylline •Antipyretics •Baclofen •Favipiravir •Levocetirizine •Montelukast •N-acetylcysteine •Oral antibiotics •Oseltamivir •Proton pump inhibitors •Short-acting insulin •Steroids •Vitamin c •Vitamin d •Zinc
Sene, 2021 (Brazil) (25)	29 (M)	NA	•↑ CRP •↑ Neutrophils •↓ Lymphocytes	•Cough •Mild SOB •Rhinorrhea	2 days	Symptoms improved after 10 hours	•Acetaminophen •Chlorpromazine
Talwar, 2021 (India) (26)	49 (M)	•Hypertension	•↑ D-dimer •↑ RR •↑ Urea •↓ Hb •↓ Na •↓ PLT	•Fever	3 days	Symptoms improved	•Dexamethasone •Ivermectin •Low molecularweight heparin •Remdesavir
	22 (F)	NA	•↑ D-dimer •↑ Urea •↓ Hb •↓ Na	NA	5 days	No improvement	•Dexamethasone •Ivermectin •Low molecular weight heparin •Metoclopramide •Remdesavir
	70 (M)	NA	•↑ CRP •↑ D-dimer •↑ Ferritin •↑ PLT •↑ Total bilirubin •↑ Urea •↓ Hb	•Weight loss	8 days	Symptoms improved	•Antibiotics •Baclofen •Dexamethasone •Ivermectin •Low molecular weight heparin •Remdesavir
Totomoch- Serra, 2021 (Chile) (27)	60 (M)	•Hypercholesterolemia	•↑ D-dimer •↑ GGT •↑ LDH •↓ Calcium •↓ Sodium •87% SPO2 (hypoxemia)	•Dysgeusia •Fever	>2 days	Symptoms improved after 72 hours	•2% lidocaine •Acetaminophen •Clonazepam •Haloperidol •Ilaprazole •Metoclopramide

*ALP, Alanine aminotransferase; ALT, Alanine aminotransferase; AST, Aspartate aminotransferase; BG, Blood glucose; Cl, Chloride CRP, C-reactive protein; FBS, Fasting blood sugar; GGT, Gamma-Glutamyl Transpeptidase; Hb, Hemoglobin; LDH, lactate dehydrogenase; Na, Sodium; PLT, Platelet; RBCs, Red blood cells; RR, Respiratory rate; WBCs, White blood cells.*

*↑, Increased; ↓, Decreased.*

* *Persistent hiccups developed after COVID-19 diagnosis*.

### Quality of the Studies

Quality assessment of the eligible studies revealed that on average all of the recommended elements were fulfilled and thus, these were considered as low risk of bias. Only two studies did not attain a perfect score. The information most commonly marked as “Not/Applicable” across the studies was drug administration reaction interface (Table 2).

**Table 2:** Quality assessment of the included studies.

**Author, year**	**Q1**	**Q2**	**Q3**	**Q4**	**Q4**	**Q5**	**Q6**	**Q7**	**Q8**
Ali, 2021 (Kenya) (16)	^•^	^•^	^•^	^•^	^•^	^◦^	^◦^	NA	^•^
Alvarez-Cisneros, 2021 (Mexico) (17)	^•^	^•^	^•^	^•^	^•^	^•^	^Ø^	NA	^•^
Atiyat, 2021 (USA) (18)	^•^	^•^	^•^	^•^	^•^	^•^	^•^	NA	^•^
Bakheet, 2021 (Egypt) (19)	^•^	^•^	^•^	^•^	^•^	^•^	^•^	NA	^•^
Chiquete, 2021 (Mexico) (20)	^•^	^•^	^•^	^•^	^•^	^•^	^•^	NA	^•^
Dorgalaleh, 2020 (Iran) (21)	^•^	^•^	^•^	^•^	^•^	^•^	^•^	NA	^•^
Ikitimur, 2021 (Turkey) (22)	^•^	^•^	^•^	^•^	^•^	^•^	^•^	NA	^•^
Karampoor, 2021 (Iran) (23)	^•^	^•^	^•^	^•^	^•^	^•^	^•^	NA	^•^
Prince, 2020 (USA) (8)	^•^	^•^	^•^	^•^	^•^	^•^	^•^	NA	^•^
Sangamesh, 2021 (India) (24)	^•^	^•^	^•^	^•^	^•^	^•^	^•^	NA	^•^
Sene, 2021 (Brazil) (25)	^•^	^•^	^•^	^•^	^•^	^•^	^•^	NA	^•^
Talwar, 2021 (India) (26)	^•^	^•^	^•^	^•^	^•^	^•^	^•^	NA	^•^
Totomoch-Serra, 2021 (Chile) (27)	^•^	^•^	^•^	^•^	^•^	^•^	^•^	NA	^•^

*NA, Not applicable; Q1, Were patient's demographic characteristics clearly described?; Q2, Was the patient's history clearly described and presented as a timeline?; Q3, Was the current clinical condition of the patient on presentation clearly described?; Q4, Were diagnostic tests or methods and the results clearly described?; Q5, Was the intervention(s) or treatment procedure(s) clearly described?; Q6, Was the post-intervention clinical condition clearly described?; Q7, Were adverse events (harms) or unanticipated events identified and described?; Q8, Does the case report provide takeaway lessons?*

• *Yes;*

◦ *No;*

Ø *Unclear*.

## Discussion

Our systematic review examined the co-occurrence of persistent hiccups with SARS-CoV-2 infection. We included 13 reports of 16 patients in which presenting complaints of hiccups were reported by hospitalized COVID-19 patients. Our findings revealed that metoclopramide, chlorpromazine, and baclofen were used as the primary treatments for managing the hiccups, while dexamethasone, azithromycin, ivermectin, and ceftriaxone, for the infection from SARS-CoV-2. After the initiation of treatment, the majority of patients showed improvement of symptoms and were later recovered in stable condition.

A hiccup is a benign self-limiting reflex most commonly affecting males (9, 10, 28). It is characterized by repetitive sudden involuntary spasmodic contractions of the diaphragm and intercostal muscles (9, 10). These contractions usually are accompanied by a short inhalation, which is interrupted by closure of the glottis (9, 10). The hiccup response appears to involve a reflex arc that arises from the interaction of three neuroanatomical facets (29–31). An “afferent limb” composed of the phrenic and vagus nerves as well as sympathetic nerve fibers from the thoracic chain T6–T12, which mediates the outflow of visceral and somatic sensory inputs. An “efferent limb” consisted chiefly of the phrenic nerve, which conveys motor commands to the diaphragm and accessory respiratory muscles of the intercostal space. A central “processing unit” involving nonspecific anatomic structures between the upper spinal cord at the mid-cervical vertebrae (at levels) C3-C5 and the brainstem, which integrates the interaction between the afferent and efferent arms.

Persistent hiccups can arise from the direct structural or functional injury of the hiccup reflex arc itself, which leads to persistent triggering but may result from any underlying disease affecting the reflex nerves involved (32). In patients with COVID-19, the manifestation of persistent hiccups appears to be a complex phenomenon, with several theories proposed. The most compelling theory describes the ability of COVID-19 related-pneumonia to cause an injury on peripheral nerves, especially the vagus or phrenic nerves, which support the diaphragm musculature and lead to its irritation (33–36). Considering that the chest X-ray or computed tomography scan of the including patient cohort demonstrated findings of interstitial pneumonia compatible with COVID-19, an inflammatory-based pneumonic irritation of the vagus or phrenic nerve distributions and their pericardial, gastric, and esophageal branches, may explain the manifestation of persistent hiccups following SARS-CoV-2 infection.

Casual of persistent hiccups may also be the administration of pharmacological agents that may exert their action *via* the stimulation of the gastrointestinal or central nervous system (CNS) (30, 37–42). Although drug-induction is not widely considered as the triggering factor for recurrent episodic hiccups, drugs, such as corticosteroids and antibiotics, are often cited as a possible source of causation (39, 43–45). Particularly, with the absence of curative treatment pertained to antiviral or immunomodulatory interventions, a surge in corticosteroid therapy, such as dexamethasone, for controlling the severity of SARS-CoV-2 infection, has been increasingly popular (46–48). As reported, persistent hiccups secondary to corticosteroid administration, which often are supplementary to primary treatment and especially upon high doses, becomes worrisome (49). Equally, a substantial pre- and post-emptive use of antibiotics especially those available without a prescription, has been documented in SARS-CoV-2 infected patients, which has now developed into a deluge driven by both patients and physicians (50–53). Although information relevant to the diagnosis of co-infection from a bacterial source was not reported nor investigated in the included patients of which a plethora received azithromycin and ceftriaxone, antibiotic induced-persistent hiccups in the setting of COVID-19 treatment warrants further attention. Overall, there is not ample proof of drug-induced persistent hiccups to propose causation, however, the incidence may be adequate enough to raise this phenomenon and its potential association with SARS-CoV-2 infection, as a concern to practitioners.

Additional extrapulmonary factors to the manifestation of persistent hiccups have also been proposed and these include encephalitis lethargica and somatoform disorder. To date, there is increasing evidence of diverse neurological cases of encephalopathy following SARS-CoV-2 infection (54–57). Evidence from the influenza pandemic points out that the association between persistent hiccups and SARS-CoV-2 infection may be etiologically related to encephalitis lethargica (58–60). Considering that SARS-CoV-2 can be neuroinvasive with an olfactory transmucosal route to the CNS, this may hint the manifestation of persistent hiccups in COVID-19 patients (60, 61). Although examination of the cerebrospinal fluid (CSF) was not ensued in the included patients, differential diagnosis of viral encephalitis is largely contingent on CSF virus detection (55, 57). While SARS-CoV-2 dissemination in the CNS has been described as transient and/or limited with low CSF titres (55, 57), reports confirm negative CSF PCR positivity with delayed neurological involvement (62). Taken together, whether persistent hiccups in SARS-CoV-2 infected patients may reflect an encephalitis lethargica-like phenotype or simply immune-mediated para-infectious encephalitis, remains unexplored.

Patients with a history of SARS-CoV-2 infection also present a significantly higher risk of comorbid somatic symptom disorders, and thus, symptoms of psychological distress (63, 64). Reports of persistent hiccups of psychogenic origin have been documented and classified under somatic autonomic dysfunction according to the International Classification of Diseases (ICD) 10th revision and somatic symptom disorder based on the Diagnostic and Statistical Manual (DSM) of mental disorders (DSM-V) Text Revision (TR) (65–67). Nevertheless, whether acute SARS-CoV-2 infection (as part of our cohort) could trigger the development of somatoform symptoms in predisposed subjects, cannot be commented with complete accuracy as psychogenic symptoms often accompany convalescence in long COVID-19 patients.

Beyond COVID-19 settings, hiccups may be a rare manifestation of electrolyte abnormalities or deficiencies, such as hyponatremia (68–72). Although a less compelling hypothesis, hyponatremia-induced persistent hiccups have been previously documented and may occur from cellular swelling and cerebral oedema in response to the osmolar shift (73). Despite that none of the included patients presented with severely attenuated serum sodium levels, hyponatremia has been speculated to result in the higher center inhibition of sympathetic outflow in the hiccup reflex arc, a theory worth further exploration (73). Persistent hiccups may also be a manifestation of abdominal myoclonus in clinical settings (74–76). In the context of COVID-19, presenting cases of different transient abdominal complaints, including abdominal myoclonus, have been described (77–79). Despite that no reports of paroxysmal episodes of severe abdominal pain prevailed amongst the included patients (74), masking of abdominal myoclonus by the manifestation of persistent hiccups cannot be excluded in SARS-CoV-2 infected patients. Lastly, gastrogenic and cardiogenic causes have been proposed, involving disturbances in principal organs, spanning from abdominal distension and gastric irritation to angina pectoris, and acute myocardial infraction (73, 80–82). However, considering that most of the included patients were free of any prevailing comorbidities with their medical history and health status precluding the possibility of prior episodes of severe disease, these organic causes appear far from the cause of persistent hiccups following SARS-CoV-2 infection.

### Strengths and Limitations

To our best knowledge, our study is the first to review the co-occurrence of persistent hiccups and SARS-CoV-2 infection. Our findings offer a comprehensive summary of the current literature and feature published data from included studies with quality assessment of increased scrutiny. However, our study is prone to limitations. A wider drawback involves the low-quality nature of case reports and series within our systematic review, which hampers the validity and interpretation of conclusions that can be attained. Especially, the underlying risk of bias in these studies and their selection remains unavoidable, as these become particularly vulnerable to the risk of misinterpretation or extrapolation. Thus, their reported findings although appealing, may not reflect the truth without underlying valid description.

## Conclusion

Patients infected with SARS-CoV-2 have a clinical presentation that is commonly described by symptoms, such as fever, dry cough, dyspnoea, and respiratory distress. Beyond respiratory symptoms, a growing number of complaints pertained to persistent hiccups have been recently reported by hospitalized COVID-19 patients. While the above presentation is still underreported, persistent hiccups can be burdensome to patients and could have a great impact on quality of life especially to those infected with SARS-CoV-2. Although there is not ample proof to propose causation, persistent hiccups as an atypical manifestation of SARS-CoV-2 infection cannot yet be excluded. Familiarity with unusual signs of SARS-CoV-2 infection possibly, such as persistent hiccups, is vital in raising awareness to clinicians about the diversity of presentations and the early recognition of the disease.

## Data Availability Statement

The original contributions presented in the study are included in the article/supplementary material, further inquiries can be directed to the corresponding authors.

## Author Contributions

PG conceived and designed the study. PG and KKT acquired and collated the data. KSK supervised the study. All authors drafted the manuscript and critically revised the important intellectual content.

## Conflict of Interest

The authors declare that the research was conducted in the absence of any commercial or financial relationships that could be construed as a potential conflict of interest.

## Publisher's Note

All claims expressed in this article are solely those of the authors and do not necessarily represent those of their affiliated organizations, or those of the publisher, the editors and the reviewers. Any product that may be evaluated in this article, or claim that may be made by its manufacturer, is not guaranteed or endorsed by the publisher.
